# Deporte y eugenesia: el proyecto de reglamentación del linao por el Club de Gimnasia y Deportes, Santiago de Chile, 1929

**DOI:** 10.1590/S0104-59702023000100017

**Published:** 2023-05-15

**Authors:** Alex Ovalle, Daniel Briones

**Affiliations:** i Profesor Asociado, Departamento de Ciencias Sociales/Universidad de La Serena. La Serena – Chile aovalle@userena.cl; ii Investigador Asociado, Centro Estudios Históricos/Universidad Bernardo O’Higgins. Santiago – Chile daniel.briones@ug.uchile.cl

**Keywords:** historia, deporte, linao, eugenesia, pedagogía, history, sport, linao, eugenics, pedagogy

## Abstract

Se presenta la fuente Proyecto de Reglamentación de Linao, elaborado por el Club de Gimnasia y Deportes, y publicado en Santiago en 1929. El folleto consiste en un discurso del doctor Luis Bisquertt y el corpus normativo del linao, juego de pelota ancestral. Su transcripción es de utilidad para el estudio histórico del deporte, la investigación sobre la modernización de tradiciones en la construcción de lo nacional, y a su vez, permite comprender los discursos pedagógicos y eugenésicos, asociados a la actividad profesional desarrollada por los primeros profesores de educación física a principios del siglo XX.

## Abstract (en)

The source presented is the Linao Game Regulation Project, prepared by the Club Gimnasia y Deportes, and published in Santiago in 1929. The brochure consists of a speech by Dr. Luis Bisquertt and the normative corpus of linao, an ancestral ball game. Its transcription is useful for the historical study of sport, and research on the modernization of traditions in the national construction. It is also useful to understand the pedagogical and eugenic discourses, associated with the professional activity developed by the first physical education teachers at the beginning of the twentieth century.

La difusión de los deportes en Chile y su organización en diversos ámbitos de la sociedad civil suscitaran, a principios del siglo XX, un debate en torno a las bondades asociadas a la cultura física y su aprovechamiento en la formación moral y corporal de la ciudadanía. Intelectuales, facultativos y pedagogos coincidieron en la objetivación de los hábitos saludables, la profilaxis social, el papel de la ejercitación y particularmente del deporte como un medio para la superación de los defectos congénitos en la población, como la debilidad, el raquitismo, el comportamiento licencioso, el alcoholismo y los trastornos nerviosos producidos por la vida moderna. La atención se puso en las teorías de “regeneración” racial predominantes en Europa que asumían para los seres humanos la materia prima susceptible de ser modificada en favor de un proyecto colectivo de prosperidad (Vallejo, Miranda, 2007; Sánchez, Riobó, 2020).

Sin embargo, los principios relacionados a “lucha por la vida” o la “supervivencia del más apto”, obtenidos del darwinismo social y la eugenesia positiva, confluyeron en el discurso con el rescate de rasgos consuetudinarios, somáticos y actitudinales constituidos históricamente. En este caso, la nación chilena “racializada” se sostuvo en el pasado “Araucano” (mapuche) como signo diferenciador ( Palacios, 1904 ), que, unido a la herencia visigoda traída por los conquistadores, perfilaba la imagen de una sociedad mestiza aguerrida, pero a su vez, civilizada, todos ellos argumentos ratificados, en lo formal, por los triunfos militares durante la segunda mitad del siglo XIX ( Mc Evoy, 2010 ; Cid, San Francisco, 2010).

El documento que presentamos a continuación, intitulado Club de Gimnasia Científica Proyecto de Reglamento de Linao, resulta una evidencia fundamental de lo señalado hasta ahora. Impreso en 1929, por la empresa litográfica “Nascimento” a solicitud de los miembros del Club de Gimnasia Científica en su quinto aniversario – de ahí en más Gimnasia y Deportes –, organismo que fue fundado en 1924 por los primeros profesores de Educación Física formados en la Universidad de Chile. La organización tuvo como referentes a Joaquín Cabezas, académico responsable por la adopción de la escuela sueca de ejercitación ( Martínez Fernández, 2017 ; Durán, 2014 ; Ovalle, Briones, 2022). Junto con el presidente, el médico y docente Luis Bisquertt, especialista en eugenesia científica, su trabajo fue sustentado, hasta la década de 1970, en la aplicación de diversas iniciativas de fomento corporal impulsadas por el Estado (Riobó, Villarroel, 2019).

El texto que ofrecemos comienza con un señero discurso, – quizás uno de los primeros entre toda la producción intelectual de Bisquertt. En sus líneas se atisban elementos extensamente profundizados en décadas ulteriores, con todo, en esa ocasión se utilizó para introducir la reglamentación del linao, un juego ancestral de pelota y contacto físico extraído de la cosmología inmersa en las comunidades de habla mapudungún ( López von Vriessen, 2013 ). Como se verá, su contenido refrenda lo señalado por Melo y Peres (2016) en cuanto a la configuración de un “ethos corporal” como resultado del ajuste entre elementos tradicionales y modernos, es decir, saberes originarios junto a la incorporación de la empiria occidental y moderna en América Latina.

En suma, la decisión de transcribir y publicar esta fuente cumple con una doble función. En primer lugar, poner a disposición de los investigadores el contenido del folleto conservado en la Biblioteca Nacional de Chile y con ello establecer una lectura crítica, abrir el campo de diálogo, y al mismo tiempo, ofrecer la posibilidad de rescatar y conservar el patrimonio documental relativo a la actividad física. Consideramos que es un aporte a la memoria sustentada en la indagación de evidencias directas ( Vilodre, 2010 ), sobre todo en un contexto de reciente conformación de los estudios históricos acerca del deporte, la educación física, el ocio y el tiempo libre desde una perspectiva cultural ( Ovalle, 2015 ).

En segundo lugar, estimamos que su publicación, tributa al análisis de elementos discursivos, sobre la circulación cultural de retóricas ideológicas inscriptas a las prácticas deportivas, tales como las percepciones relacionadas al cuerpo, los roles de género, la vinculación entre ciencia y deporte sumado a la configuración de identidades nacionales y raciales, que tuvieron lugar durante la modernización finisecular en la región.

## Consideraciones finales

¿Por qué establecer normas al linao? El proyecto elaborado no menciona las razones para adoptar su práctica, salvo el nombre, el reglamento parece ser la adaptación de una disciplina similar al balompié y al rugby. Para ello se requería una “cancha” – palabra quechua – de “ *foot-ball* ” – locución inglesa – para determinar el espacio en que treinta jugadores realizarían la partida. Si el afán del Club era dedicarse a la gimnasia y al deporte ¿Por qué elegir el basquetbol y el jiujitsu en vez del box y el fútbol que eran tan populares? Si bien ambas secciones del folleto (discurso y reglamento) parecen inconexas, existe una profunda razón que las une y que se puede colegir tras su lectura.

Por esos años, el balompié y el pugilismo generaban espectáculos comerciales multitudinarios. Eran la quintaesencia de las aptitudes físicas y viriles que competían por un triunfo pasajero (Ovalle, Briones, 2013; Ovalle, 2021 , 2019 ). Hemos de suponer que para Bisquertt, la banalidad del deporte como negocio significaba la decadencia del “bárbaro tecnificado” de Keyserling, incapaz de concebir una sociedad democrática, puesto que menospreciaba el ensalzamiento del espíritu parafraseando Ortega y Gasset. Bajo su punto de vista, el deportista preso a los triunfos y a récords, se alejaba del “super-hombre” de Nietzsche. Era menester repensar la civilización en su conjunto.

Tras la Gran Guerra, los países latinoamericanos perdieron la fe en Europa y la miraron con desdén por la incansable búsqueda de un progreso que enfrascó al mundo occidental en el exterminio bajo una fallida suposición acerca de la subsistencia del más fuerte ( Rinke, 2019 ). El antídoto, según el doctor, era “fundamentalmente biológico, eugenético y pedagógico.” Quienes debían dirigir la sociedad hacia su superación eran los profesores de educación física, profundizando en la razón científica y proporcionando, esta vez, las condiciones requeridas por las masas. Desde ese criterio, los más aptos no debían ser el centro de atención, sino que aquellos sujetos débiles – las mujeres y los niños – que, hasta ese momento, habían sido olvidados.

Parece ser que el linao era solo un remanente del pasado cultural originario, sin embargo, el directorio del Club aspiró a crear una disciplina que acercaba nuevamente a la antigua percepción “amateur” y no al deporte profesional. Inferimos, por tanto, que algún rescate cultural habría sido posible durante la época, aun cuando se ha de profundizar en otras fuentes, consideramos que este es un buen punto de arranque.

## Transcripción in extenso

### Transcripción in extenso

El Club de Gimnasia Científica (HOY GIMNASIA Y DEPORTES) Discurso del Presidente, Doctor, Bisquert, pronunciado con motivo del 5º aniversario del club Santiago 1929 Chile. Proyecto de Reglamento de Linao Elaborado por el Directorio de la Institución.

Señoras, señores:

Nació nuestro club del entusiasmo de un grupo de alumnos y ex alumnos de educación física, en cuyo seno cayó la semilla arrojada por un pedagogo eminente, Joaquín Cabezas. Cinco años de constante labor ha cumplido ya. Sigue adelante y continúa siendo aún el único en su género en el país.

Los ideales que persigue ya los conocéis. Ya los expusimos muchas veces y muchas veces comentamos los beneficios del ejercicio como agente mejorador del tipo humano. Quiero únicamente hacer notar ahora, a los que por primera vez me escuchan, que el Club de Gimnasia, fiel a su objetivo, empleados, deportistas y a quien quiera que sea, oportunidades para practicar racionalmente el ejercicio [ *sic* ]. Mantiene, a cargo de profesores titulados que trabajan con cariño y con tesón, cursos de gimnasia que funcionan en la Escuela de Educación Física, cuyo local cede gentilmente la dirección del establecimiento. Así que agradecemos cordialmente que el club tenga, puede decirse, su hogar en Nuestra Escuela.

Ha organizado el club una sección de *basketball* y organiza una de jiujitsu: fomenta la difusión del linao, hermoso deporte aborigen: y propicia la práctica de todos los deportes.

Ve en lo que a educación física femenina se refiere abriendo una brecha en el bloque de los prejuicios que entre nosotros prohíben a la mujer el cultivo de su cuerpo. Mantiene un curso femenino de gimnasia el cual como los masculinos no persigue records ni exhibiciones. Su existencia sola, constituye ya un evidente triunfo en medio de nuestra apatía que únicamente se conmueve ante el espectáculo sensacional: porque hay que decirlo en nuestro ambiente, el público sólo presta interés a los capaces de vencer en los torneos, es decir, a los fuertes a los que menos necesitan de ejercicio y a los que menos, por su escaso número posible, influirán en el mejoramiento físico del niño, de la multitud, que es precisamente lo que debe interesarnos.

Respecto de la mujer, no nos desembarazamos aún de la lápida de los prejuicios coloniales.

Cuando hace treinta años, Joaquín Cabezas, luchando trabajosamente en un ambiente hostil o indiferente introducía en Chile los elementos de la gimnasia nacional, hubo quienes abonaron públicamente la inutilidad del ejercicio en la mujer, atacando de frente su práctica. Se habló de inmoralidad y aunque hoy hayamos de sonreírnos, es verdad que el uniforme de pantalón provocó un verdadero escándalo. Hubo diarios que elevaron el grito en el cielo pidiendo la clausura del Instituto, que funcionaba entonces en la calle Arturo Prat. Otros llegaron a publicar, por aquellos años, malévolas caricaturas de la profesora señorita Guichard, con sus alumnas trepando por los cables.

Reímos hoy de los ingenuos escrúpulos de nuestros predecesores. Pero, tengamos cuidado. La evolución de ideas y costumbres no se para. Muy pronto los que nos sigan se reirán a su vez de nosotros, que harto nos falta para desarraigar la idea de que la mujer no ha de gozar del ejercicio. Lo he palpado muchas veces como médico y como jefe del Gabinete de Kinesiterapia.

La mujer que salida del colegio llega a practicar el juego, la gimnasia o el deporte, suele soportar amargas críticas, si ya no es joven recibe la amenaza del ridículo, como si no tuviera derecho o mejor dicho obligación, de cuidar de su salud y su belleza.

En la hora que corre, existen aún en Santiago colegios particulares en donde centenares de niñas son sometidas a riguroso sedentarismo; una hora semanal que a veces no la hay de mala gimnasia para los primeros cursos y ninguna para los cursos superiores, cursos formados por chicas en plena adolescencia o que aún no salen de la infancia. Si en tales colegios por los otros aspectos de la educación se encontrasen en un grado de atraso semejante ¿qué se esperaría de ellos?

En frecuentes ocasiones he visto pequeñas escolióticas y raquíticas de estos colegios propiciadores del sedentarismo, cuyas deformaciones vinieron a inquietar a sus confiados padres sólo cuando las ropas ya no lograron disimularlas ¡Qué de lágrimas y recriminaciones evitadas si una profesora de gimnasia hubiese dado a tiempo la señal de alarma!

Si se piensa que durante la tercera infancia y la pubertad para alcanzar su normal desarrollo atraviesa el organismo por un período de intensa labor constructiva y de hondas modificaciones biológicas que le hacen requerir imperiosamente del movimiento como del aire y del agua, se comprende que tal hecho significa todo un delito pedagógico y fisiológico perpetrado contra niñas indefensas a quienes la ignorancia de sus padres y maestros abandona: padres y maestros que, por otra parte, nada pueden saber del valor de la educación física, ya que el concepto sobre la materia no se da ni en la escuela, ni en el liceo, ni en la universidad.

Pero, por fin, ahora vemos a los gobernantes preocuparse de la educación física con el interés de la magnitud que el problema requiere. Pronto, confiamos, se pondrá remedio a mucho mal. Pronto se implantará la hora diaria obligatoria de gimnasia o juego en todos los colegios. Pronto se les dotará del material necesario y se hará una obra tal de divulgación que aniquile al fin tanto prejuicio impropio ya en el mundo que actualmente va naciendo. Se hará, creemos, obra de investigación y el perfeccionamiento en la enseñanza de la educación física dará sus frutos.

La dirección general del ramo ha elegido los profesores y médicos que estima más capaces, abnegados y entusiastas para emprender su gran tarea. Nosotros, simples espectadores, que a la vera del camino les miramos trabajar, debemos aplaudir, hacer ambiente y apoyar con nuestra propaganda las nuevas iniciativas que veamos. Y nuestro club, cooperando con todo el dinamismo y la pureza que ha de guardar lo nacido al calor del anhelo estudiantil, deberá ser como una antorcha que señale el buen camino, como una fuerza en tensión, siempre en vías de creación y de progreso, que produzca, que divulgue, que dé normas.

Hay quienes se alejan de la educación física porque sólo ven en ella y la prensa, más que nada, contribuye a esto asuntos de competencias, de campeones o de récords, en los que no deberían mezclarse gentes de constitución mediana o débil. Otros, porque piensan que aparte de aquello, se trata, en el fondo, sólo de asuntos de preparación de guerra. Error grave. Ignoran que el gran problema de la educación física, el problema básico es “fundamentalmente biológico, eugenético y pedagógico” y que, por consiguiente, ha de considerar ante todo al niño, a la mujer y al débil. Olvidan que la finalidad primera no es el soldado ni el récord, sino la superación del individuo en sus aspectos físico, intelectual, moral. Olvidan que la educación física, guardando siempre el equilibrio del cuerpo y del espíritu, va buscando el advenimiento, en todos los pueblos, de una raza mejor que la de hoy; la llegada a la tierra de hombres más sanos, más fuertes, más buenos que nosotros.

Por eso es que el educador y el médico han de mirar más hacia la multitud sumisa de futuras madres consumidas por la quietud y la rutina de la escuela vieja, que al brillante suceso deportivo. Por eso es que al maestro y al fisiólogo ha de interesar más la masa espectadora que el corto número de atletas luciendo los efébicos cuerpos en la cancha.

Se repite hoy por todas partes que, aparejado con este entusiasmo naciente por el cuerpo, junto a la ciega admiración por los campeones y a la loca avidez por los triunfos deportivos, se manifiesta un evidente menosprecio por las actividades espirituales, menosprecio que, según Ortega y Gasset, constituye la más notable característica de la época.

Los grandes pensadores de hoy observan que mientras el mundo se divierte en la fiesta del músculo y de la juventud, mientras la educación física se exalta, mientras la muchedumbre se afana por obtener el máximum de bienestar material, la alta espiritualidad, los ideales superiores de cultura y de justicia descienden, las posibilidades creadoras se agotan y la cultura occidental misma se derrumba. Así, la fe se debilita; vacila el arte; la ciencia pura no es mirada por la multitud con el respeto de antaño; domina como nunca el número y el tipo rápido de acción y sin cultura que determina las muchedumbres de hoy, el bárbaro tecnificado, como lo llama Keyserling, desplaza y derrota a la “élites”. Los ideales de libertad y democracia que hondamente agitaban a las masas y por los cuales la vida misma se ofrendaba, parecen ya ni emocionar siquiera. Y todo, sin que aún surjan con la nitidez deseada los valores nuevos que han de aparecer.

Si todo aquello es cierto: si es verdad, como los intuitivos afirman, que la cultura occidental se derrumba; si el observador, angustiado, no ve en la vida actual más que signos de vejez y decadencia disimulados por superficial frescura; y si es inútil oponer estéril resistencia a la trayectoria, fatal o no, del destino; pienso yo que nuestra actitud debe ser la de apresurarnos a tomar de la época lo que ésta pueda darnos; aquello que, en verdad, nos ofrece, que parece indicarnos con ese creciente entusiasmo por la juventud y la gloria del cuerpo bello y sano, entusiasmo instintivo que va emanado del fondo de las masas.

Y puede que, en realidad, la mayor significación de la época actual, aparte de la dominación de la naturaleza con las maravillas de la técnica, sea ésta: el mejoramiento, la ascensión en el sentido biológico, la renovación de las razas que crearán las culturas del porvenir.

Y entonces nuestro camino está trazado. La educación física, en primer término, lo señala “Mejoremos el material humano”. Remozémoslo. Aprovechaos en ello las adquisiciones de la ciencia con que continuamente se enriquecen la educación, la higiene y la eugenesia. Superemos al hombre mirando con preferencia a ese arcano en donde nace y se forma: la madre, en cuya fortaleza descansa el porvenir racial.

Así, nada nos reprocharemos en medio del desconcierto y decadencia actuales. Habremos aprovechado bellamente el confuso y fugaz momento que vivimos, y mientras unas generaciones se precipitan a la tumba y otras brotan a la vida habremos ido preparando la venida de ese super-hombre de mañana, creador, acaso, en todo el vigor de su valor biológico, de ignoradas y espléndidas culturas, que nosotros agotadas las posibilidades del mundo que ahora muere ya no podríamos hacer surgir.

**Proyecto de Reglamento de Linao, elaborado por el directorio del club de gimnasia científica**

Artículo 1º – Cancha – a) Dimensiones – Se usa la misma cancha de foot-ball, cuyas dimensiones son: largo: 118,87 m y ancho: 91,44 m como máximun; y como mínimun, 91,44 metros de largo por 47,72 m de ancho.

b) Campo neutral – En el centro de la cancha se trazan dos líneas es el campo neutral.

c) Puertas – En el centro de cada lado corto se coloca una puerta en la misma forma que el “goal” de foot-ball, es decir, postes fijos sobre la línea y separados 7,31 m y unidos por un travesaño a la altura de 2,43 m.

d) Área penal – a 16,46 m de los postes de las puertas se trazan líneas perpendiculares a los lados cortos y de una longitud de 16,46 m, cuyos extremos unidos por otra línea encierran el espacio llamado área penal.

Art. 2º – Pelota – Se usa la pelota de foot-ball núm. 3.

Art. 3º – Número de jugadores – Cada equipo se compone de 15 jugadores.

Art. 4º – Capitán – Los equipos son dirigidos y representados por sus respectivos capitanes, quienes son los únicos jugadores que pueden interponer reclamos ante el árbitro.

Art. 5º – Autoridades – El juego es dirigido por el árbitro, quien es ayudado en sus funciones por los dos jueces de línea y por los dos jueces de puerta, los que tienen un carácter meramente consultivo.

Los jueces de puerta tienen la vigilancia de todo el lado en que está ubicada la puerta que atienden.

Art. 6º – Finalidad del juego – El linao se juega entre dos equipos de 15 hombres, cada equipo trata de vencer. 1º, haciendo pasar uno o dos de sus jugadores posesionados de la pelota a través de la puerta de los contrarios, y 2º, haciendo pasar la pelota en su totalidad a través de la puerta de los contrarios por medio de un tiro a la puerta concedido como penalidad por determinadas infracciones de los defensores.

Art. 7º – Uniforme – Pantalón corto que no cubra las rodillas, pies descalzos o con zapatos o zapatillas con planta lisa.

Art. 8º – Duración del juego – Se juega dos períodos de 25 minutos cada uno con un descanso intermedio de 10 minutos.

Art. 9º – Reemplazo de jugadores – Hasta tres reservas pueden entrar a reemplazar jugadores que deban retirarse de la cancha por accidente, castigo o conveniencia del equipo manifestada al árbitro por el respectivo capitán.

El cambio voluntario de jugadores se hará con la autorización del árbitro durante la suspensión de juego originada por cualquier motivo.

Art. 10 – Tantos o puntos – Se marca un tanto o punto cuando uno o varios jugadores, posesionados de la pelota, pasan a través de una puerta. Los contrarios de la puerta batida se anotan un tanto o punto. También se marca un tanto cuando la pelota pasa en su totalidad una puerta a consecuencia de un tiro a la puerta.

Art. 11 – Carga – Hasta dos jugadores por equipo pueden al mismo tiempo luchar la pelota por conseguir su posesión y sólo dentro del área penal pueden actuar tres defensores, debiendo en tal caso estar incluido el portero respectivo.

Art. 12 – Iniciación del juego – Estando cada equipo dentro de su campo distribuido libremente, el árbitro lanzará la pelota al aire verticalmente para que pueda caer dentro del campo neutral, campo que puede ser invadido desde el momento que la pelota se eleva.

Art. 13 – Faltas – a) Personales – Golpear a un jugador; arrastrarlo; saltar sobre él, tocándolo; tomarlo de las piernas; torcerle los brazos o cuello; intento de juego peligroso por su violencia.

b) Técnicas – Cargar ilegalmente; poner la pelota en juego sin la orden del árbitro, excepto en el lanzamiento desde afuera; tocarla con los miembros inferiores o con la mano cerrada.

Art. 14 – Penalidades – a) En las faltas personales la pelota pasa a un jugador contrario del que cometió la falta y la pone en juego desde el mismo sitio de la infracción

b) El jugador que comete una falta personal intencional es descalificado inmediatamente por el árbitro y reemplazado por una reserva.

c) Si la falta personal o la carga ilegal es cometida por un jugador dentro de su propia área penal, se concede al equipo atacante un tiro a la puerta.

Art. 15 – Tiro a la puerta – El tiro a la puerta se sirve desde una marca situada a 13 metros de la línea de la puerta, frente al centro de ésta, debiendo la pelota ser impulsada con una o ambas manos.

Al iniciarse el tiro sólo estarán dentro del área penal el portero y el atacante que sirve el tiro, y ningún jugador podrá entrar al campo indicado mientras la pelota no toque la tierra, los postes de la puerta o el portero. Si el infractor a esta regla es atacante y se produce el tanto, éste se anula y la pelota es considerada fuera de la cancha; si es defensor, se hace válido y si no se marca se repite el tiro.

Art. 16 – Lanzamiento desde afuera – Cuando la pelota es echada afuera de la cancha por las líneas largas o de toque es puesta en juego por contrario del que la echó, sin necesidad de la orden del árbitro y dentro de un tiempo de 5 segundos.

Cuando un jugador saca la pelota por la línea de su propia puerta se aplica la misma penalidad anterior.

Art. 17 – Condiciones de los lanzamientos – En los lanzamientos libres y desde afuera de la cancha ningún jugador estará a menos de 4 metros del que ejecuta el lanzamiento.

Los lanzamientos libres deben efectuarse dentro de un tiempo de cinco segundos contados desde el silbato del árbitro. Y los lanzamientos desde afuera dentro de los cinco segundos contados desde el momento en que el jugador toma colocación con la pelota detrás de las líneas de límites.

Art. 18 – Lanzamiento desde la puerta – La pelota es puesta en juego por un lanzamiento desde la puerta ejecutado por el portero cuando sale de la cancha por un lado corto impulsada por un atacante, o en un tanto obtenido de un tiro a la puerta y anulado por infracción de un atacante.

Art. 19 – Pelota al centro – La pelota es puesta en juego desde el centro de la cancha al comenzar cada tiempo, después de marcado un tanto y después del segundo tiro a la puerta como penalidad de una doble falta personal.

Art. 20 – Doble falta personal – Una doble falta personal es aquella cometida por los jugadores contrarios al mismo tiempo y se castiga con un tiro a cada puerta, y si ha sido intencional, con el retiro inmediato de la cancha de los infractores.

Art. 21 – Lanzamiento vertical – En la doble falta técnica o imposibilidad para determinar el equipo que debe hacer el lanzamiento libre, la pelota se pone en juego lanzándola verticalmente hacia arriba entre dos jugadores contrarios, quienes se colocan dando la espalda a su respectiva puerta y separados uno de otro por una distancia no inferior a un metro.

Art. 22 – Lanzamiento vertical sobre la línea penal – El lanzamiento vertical entre dos por infracción dentro del área penal, se aplicará sobre el lado largo de dicha área situado frente a la puerta.

IMPRENTA NASCIMENTO

Ahumada 125. – Santiago. 192
